# Effects of high levels of CO2 on gene expression in two different genotypes of *Fagus sylvatica*

**DOI:** 10.1186/1753-6561-5-S7-P171

**Published:** 2011-09-13

**Authors:** Donatella Paffetti, Olbrich Maren, Matthias Fladung, Dieter Ernst, Thorsten Markussen, Manfred Forstreuter, Francesca Donnarumma, Veronika Kučerová, Maik Veste, Cristina Vettori

**Affiliations:** 1 Department of Agricultural and Forest Economics, Engineering, Sciences and Technologies, University of Florence, Via San Bonaventura 13, 50145 Florence, Italy; 2 Helmholtz Zentrum München, - Institute of Biochemical Plant Pathology, Neuherberg, Germany; 3 Johann Heinrich von Thuenen Institute (vTI), Institute of Forest Genetics, Sieker Landstr. 2, D-22927 Grosshansdorf, Germany; 4 Freie Universitaet Berlin, Institut fuer Biologie, Altensteinstr. 6, D-14195 Berlin, Germany; 5 Plant Genetics Institute, CNR, Via Madonna del Piano 10, I-50019 Sesto Fiorentino (FI), Italy; 6 Technical University in Zvolen, T.G. Masaryka 24, SK-96053 Zvolen, Slovakia; 7 Centre for Energy Technology Brandenburg (CEBra), Bioenergy Research, 03046 Cottbus, Germany

## Background

The scenario of a changing environment, widely termed as global change is mainly caused by human activities. Oil and carbon combustion, the use of chlorofluorocarbons, and deforestation are one of the main factors responsible for increasing CO_2_ concentration and for an increase of air temperature. In addition global changes will affect precipitation patterns, nitrogen concentration in the atmosphere and enhanced UV-B radiation. Forest trees constitute a relevant economic and ecological resource that is under severe pressure by such environmental changes.

However, the response of forest trees and in particular of the important forest tree species *Fagus sylvatica* to elevated CO_2_ levels on a gene expression basis is unknown so far. The principal aim of this study is the investigation of two different genotypes of *Fagus sylvatica* upon increased CO_2_ by microarray and gene expression analyses.

## Materials and methods

Shoots of *F. sylvatica* (Germany) and *F. sylvatica* “purpurea tree” (Germany) were grafted on *F. sylvatica* rootstocks. Plants were kept for 10 days under controlled conditions in climate chambers using the same temperature and light parameters, whereas the CO_2_ concentration was approx. 380-400 ppm (ambient) in the control chamber and 1000 ppm (high) in the CO_2_ chamber. Five leaves were taken from arbitrary chosen plants in control and high CO_2_ chamber at 2 different time points (T2=2 days and T10=10 days) with 3 biological replicatesper each genotype per each sampling time point. The leaves were immediately frozen in liquid nitrogen and stored at -80°C. Extraction of total RNA was according to Kiefer *et al*[1] and microarray analyses were carried out as described by Olbrich *et al*[2]. Real Time PCR (RT-PCR) [3,4] analyses were carried out for selected genes to evaluate changes in gene expression.

## Results

The microarray resulted in differentially expressed gene at T2 and T10 in each beech genotype analyzed. In particular, genes involved in photosynthesis and chloroplast biogenesis were up regulated at T2. A similar trend was been for genes involved in sugar metabolism. This indicates that, at high CO_2_ concentration, the activity of the photosynthesis machinery will be accelerated and, therefore, also the one of the glycolysis pathway which brings to an accumulation of carbon. After 10 days of high CO_2_ level, few of these genes were down regulated but most of them are expressed at normal level. This effect has previously been described in most of the studies on pot-grown other C3 plants under elevated CO_2_ which have indicated photosynthetic acclimation [5]. This response was particularly evident for the *F. sylvatica*“purpurea tree” showing a different behaviour to the second beech genotype.

RT-PCR for genes involved in photosynthesis, chloroplast biogenesis and sugar metabolism are in progress for both genotypes.

## Conclusions

A lot of studies have reported contradictory responses of higher C3 plants to elevated levels of CO_2_. This observation has also been made in the two German beech genotypes analyzed in this work confirming high expression variability between different genotypes of the same plant species. Therefore, considering the importance to preserve the forest ecosystems under global climate change, further investigations are necessary to understand the regulatory events associated with the adaptive acclimation responses of trees.

A lot of studies have reported contradictory responses of higher C3 plants to elevated levels of CO_2_. This observation has also been made in the two German beech genotypes analyzed in this work confirming high expression variability between different genotypes of the same plant species. Therefore, considering the importance to preserve the forest ecosystems under global climate change, further investigations are necessary to understand the regulatory events associated with theadaptive acclimation responses of trees.
